# Care for the Eyes

**Published:** 1875-12

**Authors:** D. F. Lincoln


### CARE OF THE EYES.

BY D. F. LINCOLN, M. D.

When writing, reading, drawing, sewing, etc., always take care that the room is comfortably cool and the feet warm; that there is nothing tight about the neck; that there is plenty of light without dazzling the eyes. that the sun does not shine on the object we are at work upon; that the light does not come from the front; it is best when it comes over the left shoulder; that the head is not bent very much over the work; that the page is nearly perpendicular to the line of sight; that is, that the eye is nearly opposite the middle of the page, for an object held slanting is not seen so clearly; that the page or the object, is not less than fifteen inches from the eye. Near-sightedness is apt to increase rapidly when a person wears, in reading, the glasses intended to enable him to see distant objects. In any case, when the eyes have any defect, avoid fine needlework, drawing of fine maps, and all such work, except for very short tasks, not exceeding half an hour each, and in the morning. Never study or write before breakfast by candle-light. Do not lie down while reading. If your eyes are aching from fire-light, from looking at the snow, from over-work, or other causes, a pair of colored glasses may be advised to be used for a while. Light blue or grayish blue is the best shade, but these glasses are likely to be abused, and usually, are not to be worn except under medical advice. Almost all who continue to wear colored glasses, having perhaps first received advice to wear them from medical men, would be better without them. Traveling venders of spectacles are not to be trusted; their wares are apt to be recommended as ignorantly and indiscriminately as in the times of the "Vicar of Wakefield." If you have to hold the pages of *Hall's Journal* nearer than fifteen inches in order to read it easily, it is probable that you are quite near-sighted. If you have to hold it two or three feet away before you see easily, you are probably far sighted. In either case, it is very desirable to consult a physician before getting a pair of glasses, for a misfit may permanently injure your eyes. Never play tricks with the eyes, such as squinting or rolling them. The eyes are often troublesome when the stomach is out of order. Avoid reading or sewing by twilight, or when debilitated by recent illness, especially fever. Every seamstress ought to have a cutting-out table, to place her work on such a plane with reference to the line of vision as to make it possible to exercise a close scrutiny without bending the head or figure much forward. Usually, except for aged persons or chronic invalids, the winter temperature in workrooms ought not to exceed 60 or 65 degrees. To sit with impunity in a room at a lower temperature, some added clothing will be necessary. The feet of a student or seamstress should be kept comfortably warm while tasks are being done. Slippers are bad. In winter the temperature of the lower part of the room is apt to be 10 or 15 degs. lower than that of the upper. It is indispensible in all forms of labor requiring the exercise of vision of minute objects, that the worker should rise from his task now and then, take a few deep inspirations with closed mouth, stretch the frame out into the the most erect posture, throw the arms backward and forward, and, if possible, step to a window or into the open air, if only for a few moments. Two desks or tables in a room are valuable for a student; one to stand at, the other to sit at.

